# A Functional Nuclear Localization Sequence in the *C. elegans* TRPV Channel OCR-2

**DOI:** 10.1371/journal.pone.0025047

**Published:** 2011-09-21

**Authors:** Meredith J. Ezak, Denise M. Ferkey

**Affiliations:** Department of Biological Sciences, State University of New York at Buffalo, Buffalo, New York, United States of America; Yale University, United States of America

## Abstract

The ability to modulate gene expression in response to sensory experience is critical to the normal development and function of the nervous system. Calcium is a key activator of the signal transduction cascades that mediate the process of translating a cellular stimulus into transcriptional changes. With the recent discovery that the mammalian Ca_v_1.2 calcium channel can be cleaved, enter the nucleus and act as a transcription factor to control neuronal gene expression, a more direct role for the calcium channels themselves in regulating transcription has begun to be appreciated. Here we report the identification of a nuclear localization sequence (NLS) in the *C. elegans* transient receptor potential vanilloid (TRPV) cation channel OCR-2. TRPV channels have previously been implicated in transcriptional regulation of neuronal genes in the nematode, although the precise mechanism remains unclear. We show that the NLS in OCR-2 is functional, being able to direct nuclear accumulation of a synthetic cargo protein as well as the carboxy-terminal cytosolic tail of OCR-2 where it is endogenously found. Furthermore, we discovered that a carboxy-terminal portion of the full-length channel can localize to the nucleus of neuronal cells. These results suggest that the OCR-2 TRPV cation channel may have a direct nuclear function in neuronal cells that was not previously appreciated.

## Introduction

The transient receptor potential (TRP) family of ion channels can be found in all eukaryotes from yeast to mammals [1], [2]. TRP channel proteins contain 6 transmembrane domains and are thought to assemble as homo- or hetero-tetramers to form cation selective channels [1], [3]. The commonly accepted nomenclature subdivides TRP proteins into 7 subfamilies: TRPA, TRPC, TRPM, TRPML, TRPN, TRPP and TRPV [1], [3], [4]. TRP channels are activated by a wide range of stimuli, including intra- and extracellular messengers, chemicals, mechanical forces and osmotic stress [1], [3] and they have been linked to many different sensory modalities including vision, taste, olfaction, hearing and touch [2], [3]. Although the different TRP subfamilies have diverse activation properties and functions, all TRP channels share the unifying characteristic of being permeable to calcium [4].

Mammalian TRP vanilloid family (TRPV) channels range from modestly to highly calcium permeable and contain 3–5 cytoplasmic amino-terminal ankyrin repeats as well as a long, unconserved carboxy-terminal cytoplasmic tail [1], [5]. TRPVs are widely expressed in mammals, but most thoroughly studied in sensory neurons [4]. While they are best known for their thermosensitivity, with 4 of the 6 members being activated by heat, TRPVs are also broadly involved in nociception and are activated by a variety of physiologically important cues including osmotic cell swelling, noxious chemicals, analgesic compounds, inflammatory cytokines, calcium store depletion and polyunsaturated fatty acids (PUFAs) [1], [3], [4]. Furthermore, most TRPVs are polymodally activated, allowing them to integrate signals from multiple cellular pathways [3].

The *C. elegans* genome encodes members of all seven TRP subfamilies, including 5 TRPVs (OSM-9, OCR-1, OCR-2, OCR-3 and OCR-4) [2], [5], [6]. Each *ocr* gene is only expressed in a subset of cells that express OSM-9, suggesting that the individual OCR channel subunits usually function together with OSM-9 [5]. *C. elegans* TRPV channels have been well studied for their role in olfaction and nociception [2], [5], [7], [8], [9], [10]. OSM-9 and OCR-2 are expressed together in the sensory cilia of AWA and ASH head sensory neurons, where it is believed that they promote each other's proper localization to the dendritic cilia to function in sensory transduction [5]. Loss of OSM-9 or OCR-2 results in mild to severe defects in AWA-mediated olfactory responses and ASH-mediated nociceptive avoidance behaviors [7], [8], [9], [10].

Beyond their roles in sensory signaling, OSM-9 and OCR-2 also regulate the transcription of sensory genes. *osm-9* and *ocr-2* mutant animals have reduced expression of ODR-10, a G protein-coupled receptor expressed in the AWA olfactory neurons [5], as well as the serotonin biosynthetic enzyme TPH-1 in the ADF chemosensory neurons [11]. While the precise molecular mechanism by which OSM-9/OCR-2 controls expression of these downstream targets is unknown, it has been proposed that OSM-9/OCR-2 function in activity-dependent gene expression pathways to regulate *odr-10* and *tph-1* expression [5], [11]. First discovered almost 3 decades ago, activity-dependent regulation of transcription is a cellular mechanism whereby a transient signaling event results in long-lasting changes in gene expression [12], [13], [14]. This process is particularly important in neuronal cells as it ensures the proper development and maturation of synaptic connections and allows for adaptations within the adult nervous system that underlie learning and memory [15], [16]. Mammalian genome-wide analysis has revealed over 300 genes that are transcriptionally regulated in response to neuronal activity [15], [16], [17].

Studies of mammalian activity-dependent genes have revealed that most transcriptional changes occur downstream of calcium entry into the cell either through plasma membrane channels or by release of calcium from internal stores [15], [18]. Typically, this influx activates a number of signal transduction cascades, such as Ca^2+^/calmodulin-dependent protein kinases and Ras/MAPK pathways, that converge to modify nuclear transcription factors, such as CREB, and regulate expression of activity-dependent genes [15], [16], [19], [20], [21], [22], [23]. However, a new mechanism directly linking calcium channels to transcriptional regulation was recently discovered. Gomez-Ospina et al. (2006) reported that a carboxy-terminal fragment of the mammalian Ca_v_1.2 L-type voltage-gated calcium channel localizes to the nucleus, functions as a transcription factor, and directly regulates expression of a number of neuronal genes [24]. This new mode of regulation bypasses calcium-activated signaling cascades, previously thought to be the sole means connecting calcium channels to transcription, and was the first example of a calcium channel functioning in the nucleus as a transcription factor.

In this study, we report the discovery of a bipartite nuclear localization sequence (NLS) in the cytoplasmic carboxy-terminus of the *C. elegans* TRPV cation channel OCR-2. NLSs are stretches of amino acids that mediate the active transport of proteins into the nucleus [25]. We find that the OCR-2 NLS is functional, being both sufficient for the nuclear import of a GFP fusion in neuronal cells and able to direct nuclear accumulation of a carboxy-terminal cytoplasmic fragment of the OCR-2 channel. Key basic residues required for mammalian NLS function are also required for the OCR-2 NLS to direct nuclear localization. Furthermore, while our investigation into the subcellular localization of the full-length OCR-2 channel supports previous reports of expression in neuronal cell bodies and cilia, we also discovered that a portion of the full-length channel can localize to the nucleus in both head and tail neurons.

## Results

### The Carboxy-Terminal Tail of OCR-2 Contains a Bipartite Nuclear Localization Sequence

**Figure 1:**
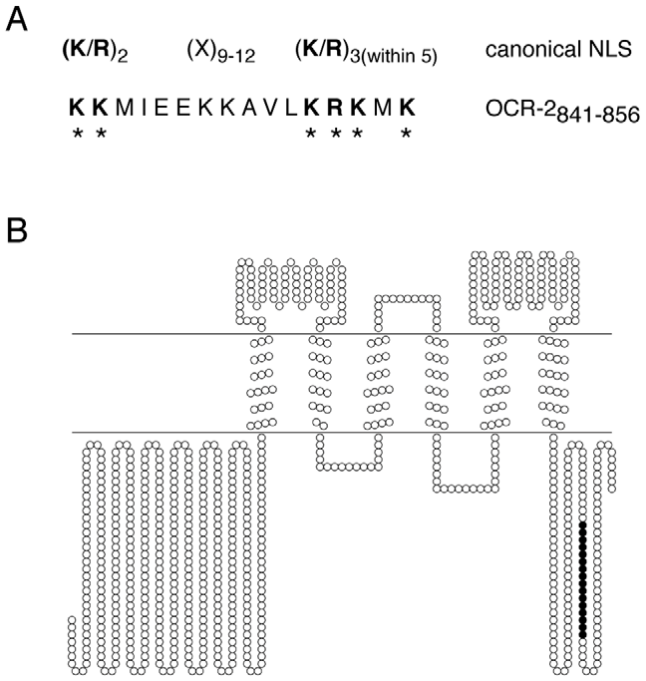
A putative bipartite NLS in the TRPV channel OCR-2. Amino acids 841–856 of OCR-2 contain two clusters of lysine/arginine residues, separated by 9 amino acids. (A) Alignment with the canonical bipartite nuclear localization sequence demonstrates that the OCR-2 sequence closely matches an NLS. (B) A diagram of the *C. elegans* OCR-2 channel, with the putative NLS in black, reveals that the sequence is located in the cytoplasmic carboxy-terminus of the channel. The cytoplasmic side is located below the horizontal lines that denote the plasma membrane. (Diagram was created using TOPO2, Transmembrane Protein Display software, http://www.sacs.ucsf.edu/TOPO2).

**Figure 2:**
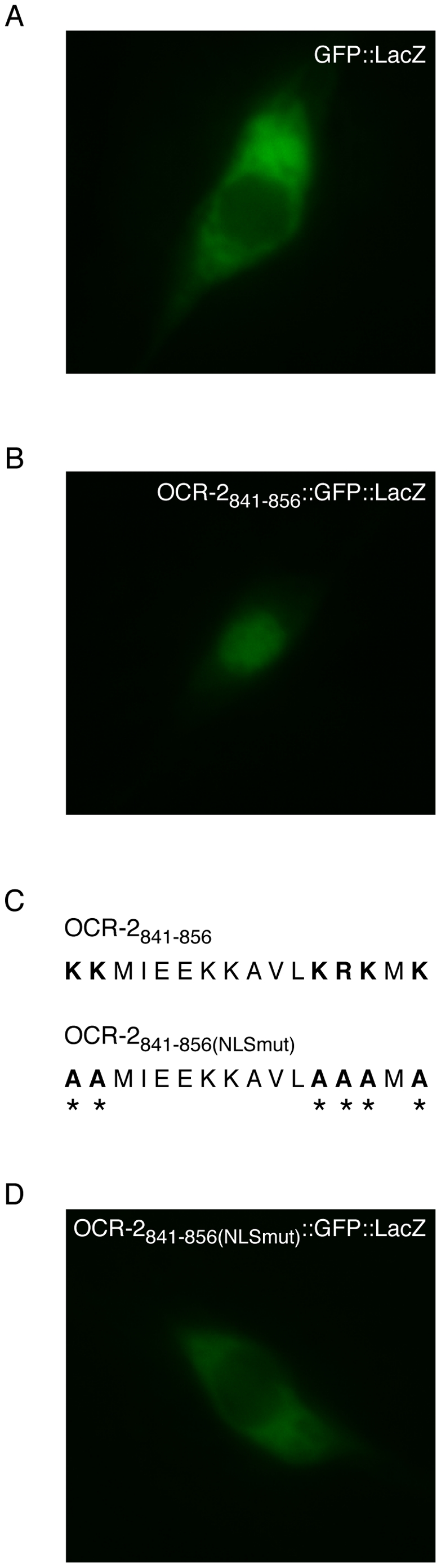
The putative NLS in OCR-2 is sufficient to drive nuclear localization of a GFP::LacZ fusion protein, and requires basic residues within the NLS. The *osm-10* promoter [30] was used to express GFP::LacZ fusion proteins in the ASH neurons. (A) The GFP::LacZ fusion (*osm-10p::gfp::lacZ*) alone is excluded from the nucleus of the ASH neurons. GFP fluorescence was restricted to the cytoplasm in 78/78 animals assessed. (B) Inclusion of the putative NLS, upstream of and in frame with GFP::LacZ (*osm-10p::ocr-2_841–856_::gfp::lacZ*), resulted in robust nuclear translocation of GFP. The ASH neurons of 97/103 worms examined displayed pronounced nuclear accumulation of GFP. (C) Six of the basic lysine and arginine residues (indicated in bold) were replaced with the nonpolar amino acid alanine (indicated with an asterisk and in bold) and the mutated NLS was fused upstream of and in frame with GFP::LacZ (*osm-10p::ocr-2_841–856(NLSmut)_::gfp::lacZ*). (D) Mutating these basic clusters abolished nuclear localization. The OCR-2_841–856(NLSmut)_::GFP::LacZ fusion was restricted to the cytoplasm of ASH in 44/44 animals examined. Numbers represent the combined data of ≥3 independent transgenic lines for each construct.

Canonical bipartite NLSs contain two clusters of basic residues, separated by a spacer region of 9–12 amino acids [25], [26]. Using the Expasy Prosite Protein Domain Database (http://ca.expasy.org/prosite/), we identified a putative bipartite NLS spanning residues 841–856 of the *C. elegans* TRPV channel OCR-2 that closely matches the canonical NLS sequence (Figure 1A). The NLS resides in the cytoplasmic carboxy-terminal tail of the channel (Figure 1B). To establish whether this putative NLS is able to direct nuclear transport of a cargo protein, we generated a GFP::LacZ fusion (using OCR-2 residues 841–856) to visualize subcellular localization in live animals. The inclusion of LacZ ensured that any nuclear accumulation observed was due to active nuclear import through the nuclear pore complex, rather than passive diffusion into the nucleus. Although GFP alone is small enough to passively diffuse into the nucleus, the fusion to LacZ creates a protein that greatly exceeds the 40–60 kDa passive diffusion limit of the nucleus [27], [28], [29]. We used the *osm-10* promoter [30] to drive expression of the GFP::LacZ or OCR-2_841–856_::GFP::LacZ fusion proteins in the ASHs, a pair of head sensory neurons where the endogenous OCR-2 channel is expressed [5], and observed subcellular localization via GFP fluorescence. In the absence of the putative OCR-2 NLS, the GFP::LacZ fusion was excluded from the nucleus (Figure 2A). However, inclusion of the putative NLS resulted in dramatic nuclear accumulation of the fusion protein (Figure 2B). We conclude that the OCR-2 NLS is sufficient to direct nuclear import of a cargo protein.

### Basic Residues within the OCR-2 NLS are Necessary for Nuclear Localization

The basic residues of canonical NLSs are critical for interactions with importin-α, an adaptor protein that mediates the first step of import through the nuclear pore complex [31], [32], [33]. The 2 clusters of basic residues in a bipartite NLS interact with the minor and major binding pockets of importin-α, which initiates the import of the NLS-containing protein into the nucleus [32]. To determine whether the 2 patches of basic residues within the putative OCR-2 NLS were required for the observed nuclear localization of OCR-2_841–856_::GFP::LacZ, we performed site directed mutagenesis, converting 6 basic residues within the NLS to alanine (Figure 2C). The resulting OCR-2_841–856(NLSmut)_::GFP::LacZ fusion failed to accumulate in the nucleus (Figure 2D). This suggests that, like canonical NLSs, the basic patches within the OCR-2 NLS are necessary for nuclear localization of associated proteins.

### A Carboxy-Terminal Cytosolic Fragment of OCR-2 Localizes to the Nucleus and Requires the NLS for Nuclear Accumulation

**Figure 3:**
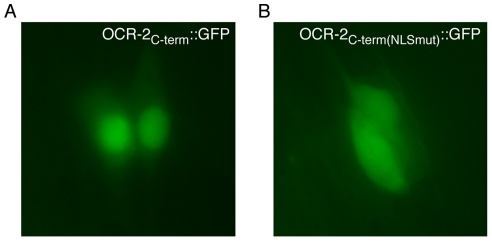
The cytoplasmic carboxy-terminal fragment of OCR-2 can utilize the NLS to localize to the nucleus. The entire carboxy-terminus (amino acids 765–900, “OCR-2_C-term_”) was fused upstream of and in frame with GFP and expressed under the control of the *ocr-2* promoter (*ocr-2p::ocr-2_C-term_::gfp*). (A) The OCR-2_C-term_::GFP fusion was detected in the nucleus of *ocr-2* expressing head and tail neurons in 91/91 transgenic animals examined. The PHA/PHB tail neurons are shown here. Furthermore, the basic residues of the NLS are required for the nuclear accumulation observed. (B) A GFP fusion to the carboxy-terminal tail in which the 6 previously identified essential lysine/arginine residues had been mutated to alanine (Figure 2C) (*ocr-2p::ocr-2_C-term(NLSmut)_::gfp*) resulted in a much less pronounced signal in the nucleus. The OCR-2_C-term(NLSmut)_::GFP signal was observed evenly distributed in the cytoplasm and nucleus of *ocr-2* expressing neurons in 94/94 transgenic animals examined. The PHA/PHB tail neurons are shown here. Numbers represent the combined data of ≥3 independent transgenic lines for each construct.

We next sought to determine whether the OCR-2 NLS could function in the context of the channel sequence. OCR-2 is a 6-pass transmembrane protein with the endogenous NLS located within the carboxy-terminal cytoplasmic tail (Figure 1B). To establish whether the carboxy-terminus of OCR-2 could localize to the nucleus, we fused GFP to the entire cytoplasmic fragment of the channel (“C-term”, residues 765–900) and expressed this fusion protein under the control of the *ocr-2* promoter, which drives expression in the AWA, ASH, ADL and ADF head neurons, as well as the PHA and PHB tail neurons [5]. In all of these neurons we observed OCR-2_C-term_::GFP accumulating to high levels within the nucleus (Figure 3A). Additionally, fusing GFP to the OCR-2 cytoplasmic tail in which the 6 basic residues of the identified NLS had been mutated to alanine (OCR-2_C-term(NLSmut)_::GFP) abolished the nuclear accumulation of GFP, and the signal was distributed evenly throughout the cell body (Figure 3B). The low-level nuclear expression seen with this fusion protein was likely due to passive diffusion into the nucleus because these fusions are relatively small, approximately 40 kDa, and may not require active transport to enter the nucleus. Furthermore, the subcellular localization pattern seen when the NLS was mutated (Figure 3B) was similar to that seen when GFP alone was expressed under the control of the *ocr-2* promoter (data not shown). As GFP lacks an endogenous NLS, this supports the notion that the low level nuclear accumulation of OCR-2_C-term(NLSmut)_::GFP observed in the absence of the functional NLS was due to diffusion. Taken together, our data indicates that the NLS can function to transport the cytoplasmic portion of the OCR-2 channel into the nucleus, and that nuclear accumulation requires the basic residues within the NLS.

### The Carboxy-Terminus of the Intact OCR-2 Channel can Localize to the Nucleus

**Figure 4:**
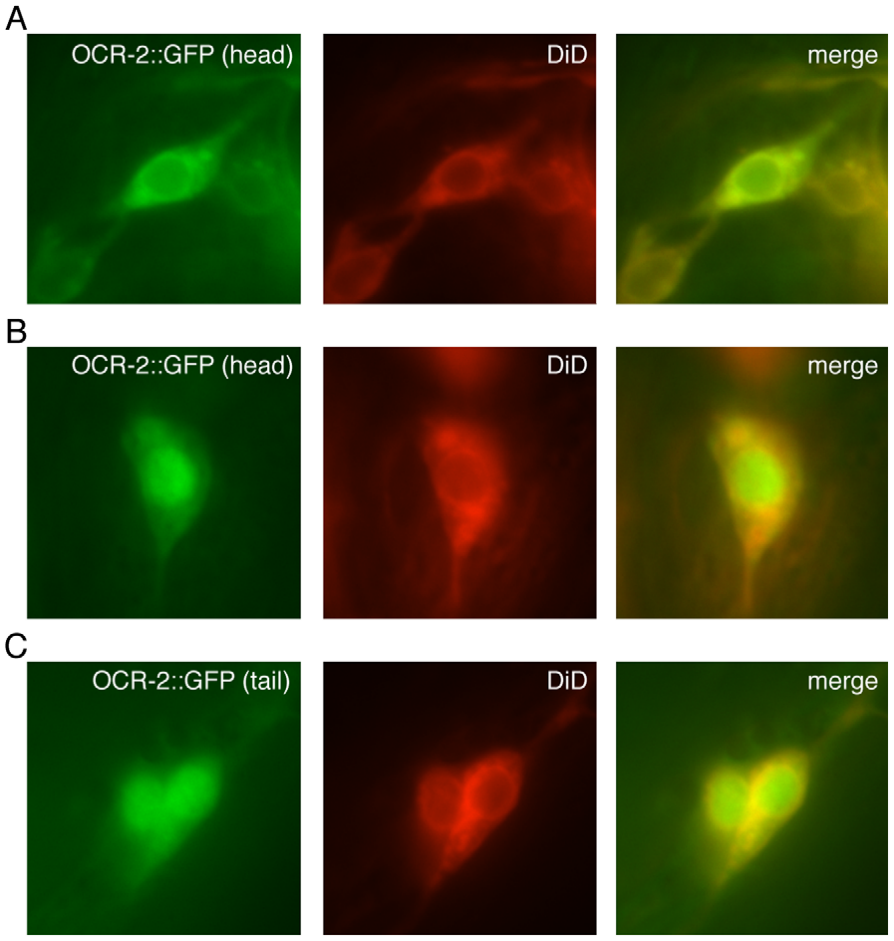
A GFP fusion to the carboxy-terminus of the full-length OCR-2 channel can be detected in the nucleus. GFP was fused downstream of and in frame with full-length OCR-2 and expressed under the control of the *ocr-2* promoter (*ocr-2p::ocr-2::gfp*). Transgenic animals were incubated with the lipophilic dye DiD (shown in red), which is taken up by several of the OCR-2 expressing neurons and was used to visualize the neuronal cell body. (A) As previously reported for an amino-terminal GFP fusion [5], flourescence was detected in the cell body and cilia of the AWA, ASH, ADL and ADF head neurons and PHA and PHB tail neurons [5]. The cell body of a head neuron is shown. (B–C) In 82/203 (40%) transgenic animals examined, distinct nuclear accumulation of GFP was detected in at least one neuron. Head (B) and tail (C) neurons are shown. Numbers represent the combined data of 3 independent transgenic lines. To show the animal-to-animal variability in subcellular localization within a transgenic line, all three images shown here were obtained from a single line.

To determine whether a portion of the full-length OCR-2 channel can translocate into the nucleus, we fused GFP to the extreme carboxy-terminus of the full-length channel. Previous characterization of OCR-2 localization in live worms made use of a cytoplasmic amino-terminal GFP fusion, and expression in neuronal sensory cilia and cell bodies was seen [5]. Consistent with reported localization, we observed GFP expression in the sensory cilia and cell body (Figure 4A and data not shown). However, with GFP fused to the carboxy-terminus of the full-length channel, we also observed nuclear accumulation of GFP in at least one *ocr-2* expressing neuron in 40% of transgenic animals (Figure 4B–C). While the majority of OCR-2 expression was seen in the cell body and the cilia, where it would fulfill its role in sensory transduction, the nuclear accumulation we observed suggests that the carboxy-terminal portion of the full-length channel is capable of translocating to the nucleus, albeit at a lower frequency.

### The OCR-2 NLS is not Necessary to Regulate *tph-1* Expression in ADF or *odr-10* Expression in AWA

**Figure 5:**
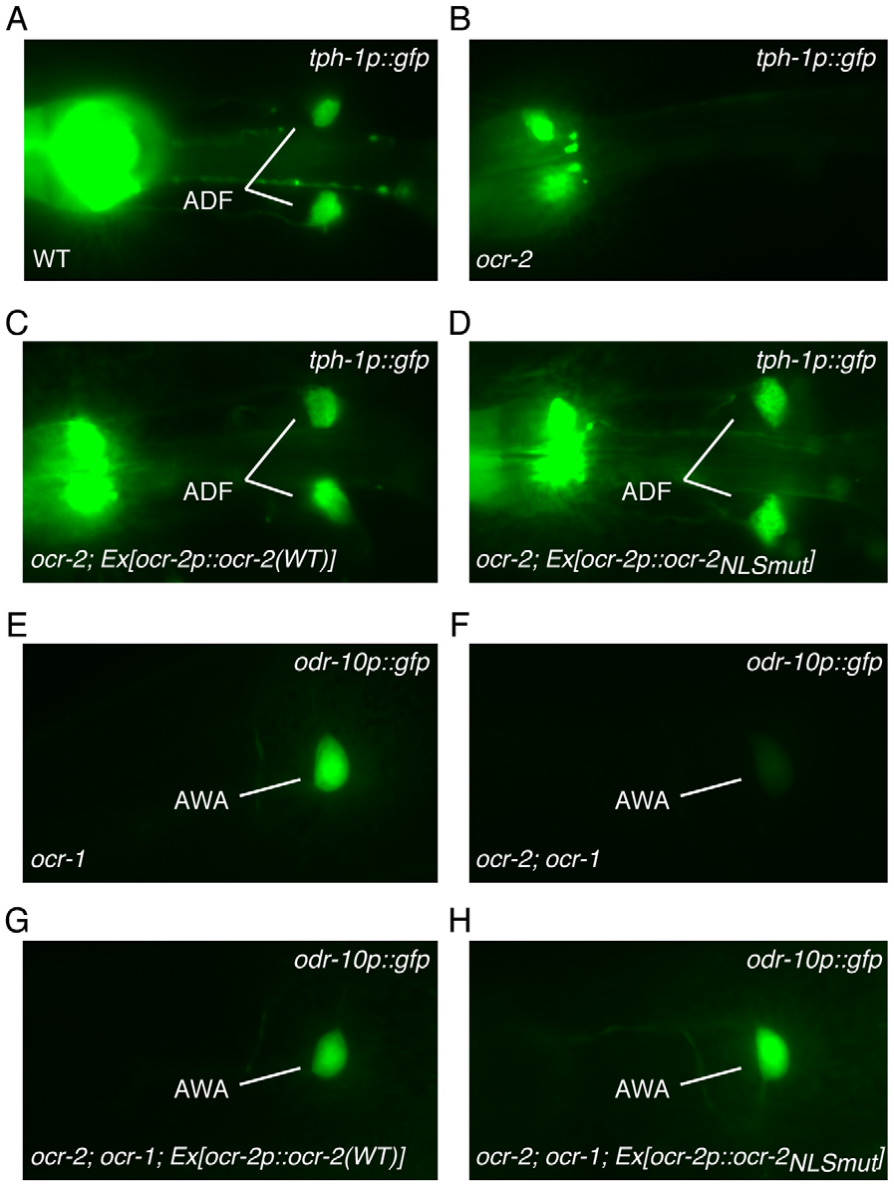
OCR-2 regulates expression of *tph-1* and *odr-10* independently of the NLS. (A–D) The OCR-2 NLS is not required for *tph-1::gfp* expression. (A) TPH-1 is a serotonin biosynthetic enzyme and, as previously described [11], a *tph-1p::gfp* transcriptional reporter is expressed in the ADF and NSM head neurons. The ADF neurons are indicated. The NSM neurons are seen in the left of each panel. (B) OCR-2 has been reported to modulate transcription of *tph-1* [11], and *ocr-2(ak47)* null mutants have reduced expression of *tph-1p::gfp* in the ADF neurons. Using the *ocr-2* promoter, either (C) the wild-type OCR-2 channel (*ocr-2p::ocr-2*), or (D) the channel in which the basic residues of the NLS had been mutated to alanine (*ocr-2p::ocr-2_NLSmut_*), was re-introduced into *ocr-2(ak47)* mutant animals. Both channels restored *tph-1p::gfp* expression in ADF. (E-H) The OCR-2 NLS is not required for *odr-10::gfp* expression. ODR-10 is a G protein-coupled receptor and an *odr-10p::gfp* transcriptional reporter is expressed solely in the AWA head neurons [5]. (E) Expression of *odr-10p::gfp* in *ocr-1(ak46)* null mutants is indistinguishable from wild-type animals (and [5]). (F) *ocr-2(ak47);ocr-1(ak46)* double mutants have drastically reduced expression of *odr-10p::gfp* (and [5]). Using the *ocr-2* promoter, either (G) the wild-type OCR-2 channel (*ocr-2p::ocr-2*), or (H) the channel in which the basic residues of the NLS had been mutated to alanine *(ocr-2p::ocr-2_NLSmut_)*, was re-introduced into *ocr-2(ak47);ocr-1(ak46)* mutant animals. Both channels restored *odr-10p::gfp* expression in AWA. ≥2 independent transgenic lines were examined for each construct, n≥40 transgenic animals.

*C. elegans* TRPV channels have previously been shown to regulate transcription of select genes in the ADF and AWA head sensory neurons [5], [11]. Both OSM-9 and OCR-2 upregulate transcription of the serotonin biosynthetic enzyme TPH-1 in the ADF neurons [11]. To assess whether the NLS of OCR-2 was required to regulate expression of *tph-1*, we used a *tph-1p::gfp* transcriptional reporter expressed in the ADF and NSM head neurons [11] (Figure 5A). Expression of GFP in the ADF neurons is dramatically reduced in *ocr-2* mutants (Figure 5B) [11]. We found that GFP expression was significantly restored in ADF by reintroducing either wild-type full-length OCR-2 (Figure 5C), or the full-length channel in which the basic residues of the NLS had been changed to alanine (OCR-2_NLSmut_) (Figure 5D).

The AWA neurons express an additional TRPV channel, OCR-1 [5]. OCR-1 and OCR-2 have overlapping functions in regulating expression of the diacetyl receptor, ODR-10, in the AWA neurons [5]. *ocr-1* mutants have no change in expression of an *odr-10p::gfp* reporter [5] (and Figure 5E), while *odr-10p::gfp* expression is only slightly reduced in *ocr-2* mutant animals [5]. However, *ocr-1;ocr-2* double mutants have little to no expression [5] (and Figure 5F). To determine whether the NLS of OCR-2 was necessary to regulate expression of *odr-10*, we used the *odr-10p::gfp* transcriptional reporter expressed solely in AWA [5]. While *ocr-1;ocr-2* double mutants have drastically reduced GFP expression (Figure 5G), we found that GFP expression was restored to levels comparable to the *ocr-1* single mutant when we reintroduced either wild-type full-length OCR-2 (Figure 5H), or the full-length channel in which the NLS had been mutated (OCR-2_NLSmut_) (Figure 5G).

Given that the OCR-2 channel that lacked a functional carboxy-terminal NLS was able to restore *tph-1p::gfp* expression in ADF and *odr-10p::gfp* expression in AWA to levels comparable to the wild-type channel, we conclude that the NLS is not necessary to regulate transcription of *tph-1* or *odr-10*. Additionally, expression of OCR-2_C-term_, under the control of the *ocr-2* promoter (*ocr-2p::ocr-2_Cterm_*), was not sufficient to restore either *tph-1p::gfp* or *odr-10p::gfp* expression (data not shown), further supporting the conclusion that nuclear localization of the OCR-2 carboxy-terminal cytoplasmic tail does not contribute to transcriptional regulation of these 2 genes. However, as these are currently the only 2 genes known to be transcriptionally regulated by TRPV channels in *C. elegans*, we can not rule out the possibility that the OCR-2 NLS is required to regulate the transcription of other, as yet unidentified, genes.

## Discussion

Transient changes in the environment can bring about long-lasting modifications in an animal's behavior by altering neural circuitry [16], [34]. This plasticity is due in part to the nervous system's ability to convert short-lived excitation into changes in gene expression, which have enduring effects. While it has long been appreciated that calcium plays a key role in coupling brief neuronal activity into long-term transcriptional changes [16], [18], [35], a direct role for the actual calcium channels involved has just begun to be appreciated [24]. The Ca_v_1.2 channel was the first calcium channel described to encode a transcription factor that directly regulates expression of a wide variety of neuronal genes [24]. TRP channels are calcium permeable channels that are involved in a wide variety of cellular processes. While members of the nematode TRPV subfamily of channels have been implicated in modulating neuronal gene expression (a novel role for TRPV channels), their precise role in this process has yet to be elucidated.

Our results demonstrate that the OCR-2 TRPV channel contains an NLS that is functional and can direct nuclear accumulation of both synthetic cargo proteins as well as the cytoplasmic portion of the native channel where it is found. Furthermore, we report that despite being a 6-pass transmembrane protein, a carboxy-terminal portion of the full-length channel is capable of accumulating in the nucleus. Previous characterization of the OCR-2 channel by other labs may have failed to detect this localization due to GFP insertions into the amino-terminus of OCR-2 [5], fixative conditions used for FLAG epitope detection [11], or because of the relatively low level frequency with which nuclear accumulation occurs.

The mechanism by which a portion of the OCR-2 transmembrane channel would be released has yet to be established. One possibility is that OCR-2 may participate in regulated intramembrane proteolysis (RIP) to release its cytoplasmic tail. RIP is a cellular process whereby a portion of a transmembrane protein is cleaved, releasing a soluble fragment that can enter the nucleus and modify gene expression, bypassing the need to activate signaling cascades in the cytoplasm [36], [37]. Since its discovery, the process has been found to be conserved from bacteria to humans and controls a wide range of cellular processes [36], [38]. While RIP has been described for surface receptors and transmembrane proteins found in the endoplasmic reticulum [39], [40], [41], [42], [43], calcium channels have only recently been added to the list of transmembrane proteins identified to undergo this type of cleavage [24]. While protease cleavage predictor programs did not reveal any obvious candidate cleavage sites within the OCR-2 channel, RIP cleavage sites may exist. Prediction programs do not use an exhaustive list of cellular proteases, and the available data for most proteases is still very incomplete. Thus, legitimate cleavage sites may go undetected.

Although the OCR-2 NLS does not seem to be required to regulate expression of the two previously identified *C. elegans* targets, *tph-1* in ADF or *odr-10* in AWA, it is possible that the TRPV channels modulate expression of other neuronal genes and that the full repertoire of genes regulated by the TRPV channels has yet to be identified. Although it has been proposed that OCR-2 also regulates gene expression in ASH [8], specific targets remain to be identified in ASH, ADL and PHA/PHB, where OCR-2 is also expressed.

We suggest that the OCR-2 channel may transcriptionally regulate neuronal gene expression both indirectly, by activating traditional calcium-dependent cascades, and directly via NLS-mediated nuclear translocation. For example, *tph-1* and *odr-10* transcripts may be regulated by calcium-mediated signaling pathways. Consistent with this possibility, a gain-of-function mutation in the Ca^2+^/calmodulin-dependent protein kinase UNC-43 partially suppresses the *tph-1p::gfp* expression defects of *ocr-2* mutants [11]. This suggests that traditional calcium-mediated signaling events downstream of OCR-2 are important, at least in part, for transcriptional regulation in the ADF neurons. While it is possible that the OCR-2 NLS has no biological role, an intriguing possibility is that the OCR-2 channel may also directly affect transcription, via nuclear localization of the carboxy-terminus, for other, yet unidentified targets in the head and tail sensory neurons. As TRP channels have already been recognized as integrators of multiple regulatory pathways, this could place TRPVs in the unique position of integrating various cellular signals and directly transducing this information to the nucleus to alter gene expression, and ultimately neuronal activity.

## Materials and Methods

### Strains

Strains were maintained under standard conditions on NGM agar plates seeded with OP50 *E. coli* bacteria [44]. Strains used in this study include: N2 Bristol wild-type, GE24 *pha-1(e2123)*, *GR1366 mgIs42[tph-1::GFP+pRF4(rol-6(su1006))]*, FG221 *ocr-2(ak47);mgIs42[tph-1::GFP+pRF4(rol-6(su1006))]*, CX3260 *kyIs37(odr-10p::gfp)*, FG231 *ocr-1(ak46);kyIs37(odr-10p::gfp)*, FG240 *ocr-2(ak47);ocr-1(ak46);kyIs37(odr-10p::gfp)*.

To create transgenic strains, germline transformations were performed as previously described [45]. For *gfp::LacZ* fusion lines, GE24 *pha-1(e2123)* animals were injected with 100 ng/ul of pFG31 *osm-10p::gfp::lacZ*, pFG36 *osm-10p*::*ocr-2_841–856_::gfp::lacZ*, or pFG41 *osm-10p::ocr-2_841–856(NLSmut)_::gfp::lacZ*, along with 150 ng/ul of pBX1 *pha-1*(+) [46] plasmid co-injection marker. For *carboxy-terminal::gfp* lines, GE24 *pha-1(e2123)* animals were injected with 50 ng/ul pFG42 *ocr-2p::ocr-2_C-term_::gfp* or pFG55 *ocr-2p:: ocr-2_C-term(NLSmut)_::gfp*, along with 75 ng/ul of pBX1 *pha-1*(+) [46] plasmid co-injection marker. For full length *ocr-2::gfp* lines, GE24 *pha-1(e2123)* animals were injected with 50 ng/ul of pFG43 *ocr-2p::ocr-2::gfp*, along with 75 ng/ul of pBX1 *pha-1*(+) [46] plasmid co-injection marker. For *tph-1p::gfp* rescue experiments FG221 *ocr-2(ak47);mgIs42[tph-1::GFP+pRF4(rol-6(su1006))]* animals were injected with 50 ng/ul of pAJ35 wild-type *ocr-2p::ocr-2* (gift of Cori Bargmann) or pFG40 *ocr-2p::ocr-2_NLSmut_*, along with 75 ng/ul of pJM67 *elt-2::gfp* [47] co-injection marker. For *odr-10p::gfp* rescue experiments FG240 *ocr-2(ak47);ocr-1(ak46);kyIs37(odr-10p::gfp)* animals were injected with 50 ng/ul of pAJ35 wild-type *ocr-2p::ocr-2* (gift of Cori Bargmann) or pFG40 *ocr-2p::ocr-2_NLSmut_*, along with 75 ng/ul of pJM67 [47] *elt-2::gfp* co-injection marker.

### Plasmid Construction

pFG40 *ocr-2p::ocr-2_NLSmut_*: The 6 basic lysine/arginine residues in the NLS were mutated to alanine using site directed mutagenesis (QuikChange, Stratagene) of pAJ35 (*ocr-2p::ocr-2*, gift of Cori Bargmann). Residue changes were verified by sequencing. In addition the entire promoter and *ocr-2* coding region was sequenced to ensure there were no unwanted base pair changes.

pFG31 *osm-10p::gfp::lacZ*: Site directed mutagenesis (QuikChange, Stratagene) was used to remove the SV40 NLS from pPD96.04 (Fire Lab *C. elegans* Vector Kit, Addgene), leaving *gfp::lacZ* and generating pFG24. The ∼900 bp *osm-10* promoter was removed from CR142 [48] using PstI and BamHI and inserted into the PstI/BamHI sites of pFG24, upstream of *gfp* and *lacZ*.

pFG36 *osm-10p*::*ocr-2_841–856_::gfp::lacZ*: The putative NLS from OCR-2 (encompassing amino acids 841–856) was PCR amplified from pAJ35 (*ocr-2p::ocr-2*, gift of Cori Bargmann) with an ATG start codon added 5′, an additional GC added to the 3′ end to maintain correct frame usage, and AgeI sites added to both the 5′ and 3′ ends. The *ocr-2_841–856_* PCR product and pFG31 were digested with AgeI and the NLS was inserted upstream of and in frame with *gfp::lacZ*. Correct orientation of the NLS was verified by sequencing.

pFG41 *osm-10p::ocr-2_841–856(NLSmut)_::gfp::lacZ*: The mutated NLS was PCR amplified from pFG40 (full length OCR-2 following site directed mutagenesis of the NLS) with an ATG start codon added 5′, an additional GC added to the 3′ end to maintain correct frame usage, and AgeI sites added to both the 5′ and 3′ ends. The *ocr-2_841–856(NLSmut)_* (mutated NLS) PCR product and pFG31 were digested with AgeI and the NLS was inserted upstream of and in frame with *gfp::lacZ*. Correct orientation of the NLS was verified by sequencing.

pFG38 *ocr-2p::gfp*: The ∼2.5 kb *ocr-2* promoter was digested out of pAJ35 (*ocr-2p::ocr-2*, gift of Cori Bargmann) with SphI and XmaI, and inserted into the SphI/XmaI sites of pPD49.26 (Fire Lab *C. elegans* Vector Kit, Addgene) to generate pFG35. *gfp* was PCR amplified from pPD95.77 (Fire Lab *C. elegans* Vector Kit, Addgene) with KpnI and SacI sites added 5′ and 3′, respectively. *gfp* was inserted into the KpnI/SacI sites of pFG35, downstream of the *ocr-2* promoter.

pFG42 *ocr-2p::ocr-2_C-term_::gfp*: The carboxy-terminus of *ocr-2*, starting with CGTACATATGAA, was PCR amplified from pAJ35 (*ocr-2p::ocr-2*, gift of Cori Bargmann). An ATG start codon was added 5′, the TGA stop codon was not included, and NheI and KpnI sites were added 5′ and 3′, respectively. pFG38 was digested with NheI and KpnI and the *ocr-2* carboxy-terminus was inserted into these sites, upstream of and in frame with *gfp*.

pFG55 *ocr-2p:: ocr-2_C-term(NLSmut)_::gfp*: The carboxy-terminus of *ocr-2* was PCR amplified from pFG40 (full length OCR-2 following site directed mutagenesis of the NLS). An ATG start codon was added 5′, the TGA stop codon was not included, and NheI and KpnI sites were added 5′ and 3′, respectively. pFG38 was digested with NheI and KpnI and the *ocr-2* carboxy-terminus with the mutated NLS was inserted into those sites, upstream of and in frame with *gfp*.

pFG43 *ocr-2p::ocr-2::gfp*: Full length *ocr-2* was PCR amplified from pAJ35 (*ocr-2p::ocr-2*, gift of Cori Bargmann). XbaI and KpnI sites were included 5′ and 3′, respectively, and the TGA stop codon was not included. pFG38 was digested with NheI and KpnI and *ocr-2* was inserted upstream of and in frame with *gfp*.

pFG52 *ocr-2p::ocr-2_C-term_*: The carboxy-terminus of *ocr-2*, starting with CGTACATATGAA, was PCR amplified from pAJ35 (*ocr-2p::ocr-2*, gift of Cori Bargmann). An ATG start codon was added 5′ and NheI and KpnI sites were added 5′ and 3′, respectively, for insertion into these sites of pFG35 (see above).

All constructs were verified by sequencing when appropriate.

### Imaging

To identify the cell body of dye-filling neurons, animals were incubated with the lipophilic dye DiD (Molecular Probes, Invitrogen), as previously described [49]. Images were obtained with a Zeiss Axio Imager Z1 microscope (using a 100× Plan-APO oil objective and epi-flourescence), high resolution AxioCam MRm digital camera and Zeiss AxioVision software.
